# Serial integration of Dean-structured sample cores with linear inertial focussing for enhanced particle and cell sorting

**DOI:** 10.1063/1.5038965

**Published:** 2018-07-09

**Authors:** Paul M. Holloway, Jonathan Butement, Manjunath Hegde, Jonathan West

**Affiliations:** 1 Centre for Hybrid Biodevices and Cancer Sciences, Faculty of Medicine, University of Southampton, *Southampton* SO17 1BJ, United Kingdom; 2 Biopharm Molecular Discovery, GlaxoSmithKline Medicines Research Centre, Stevenage SG1 2NY, United Kingdom

## Abstract

In this contribution, a channel aspect ratio of >2 was used to access high velocity regimes to provide confined sample cores by Dean focussing in advance of linear inertial focussing. This produces a singular separation origin with a mirrored transport path for efficient particle and blood cell sorting, while also increasing the spatial resolution for multiscale sorting.

## INTRODUCTION

Size sorting particulates into monodisperse fractions is a technical capability serving many aspects of biomedical research and with extension to industrial contexts. The dimensions and determinism of laminar flow makes microfluidics suitable for microparticle processing, but until recently, the throughput, typically Hertz, was inadequate for real world application. The recent introduction of inertial microfluidics,^1,2^ an approach requiring high velocity confined transport, provides the means to dramatically extend throughput (kHz–MHz). With inertial microfluidics, particles become focussed to symmetric equilibrium positions where the size-dependent shear gradient lift force balances the wall-lift force.^3,4^ The shear gradient lift force scales with velocity and relative channel dimensions, with focussing emergent as velocities approach the high, metres-per-second, regime where the benefits of extreme throughput also emerge.

The particle Reynolds number *Re_p_* is commonly used to describe particle-laden flows and involves the relative inertial and viscous forces. The *Re_p_* relates particle and channel dimension in the context of the underlying flow field and is described by *Re_p_ = Re*(*a/D_h_*)^2^, where *a* is the particle diameter, *D_h_* is the hydraulic diameter (*2hw*/(*h + w*)), and *Re* is the Reynolds number, *Re = ρŪD_h_/μ*, where *ρ* is the fluid density, *Ū* is the mean fluid velocity, and *μ* is the dynamic viscosity. Traditional microfluidics, at slower velocities, involves *Re_p_* ≪ 1, in which the particle does not disturb the underlying flow field. In inertial flow regimes, *Re_p_* tends to unity and above where particle-fluid coupling exists producing the shear gradient lift and wall-lift forces to focus particles.

A variety of inertial microfluidic circuits have been reported and comprehensively reviewed.^2,5–7^ Designs range from simple linear systems to more complex systems with secondary flows introduced using curved channel arrangements or abrupt expansion–contraction elements. Fractionation scales with particle diameter (*a*). In linear channels, focussing efficiency scales with *>a*, while in curved channels, focussing efficiency scales with *a^2^* and in expansion-contraction systems, focussing efficiency scales with *≥a^3^*, thus theoretically providing improved separation of different sized particles.^6^ Curved systems also benefit from larger lateral channel dimensions enabling higher throughput and are therefore commonly favoured. However, in practice and as with linear systems, curved systems attain modest focussing efficiency and associated purity. Additionally, inertial fractionation has limited resolving power, often restricting separations to the bimodal discrimination of highly disparate particle sizes.^8^ Thus new inertial microfluidic embodiments are needed to improve efficiency while also delivering the resolution for the separation of a range of different-sized particles and cells.

Cell sorting involves cell diameters typically restricted to a finite 2–20 *μ*m window that are represented by whole blood that contains miniature platelets, small red blood cells (RBCs), and larger white blood cells (WBCs). Using blood as a model sample, we have turned our attention to linear channels, revisiting the generalized assumptions describing the operational boundaries. We experimentally support the general relative dimensions and velocity dependencies. Our findings also demonstrate that the mean flow velocity and associated *Re_p_* window suitable for lateral focussing (without losses to vertical equilibria) is sensitive to the channel aspect ratio and can be substantially extended. Importantly, increasing mean flow velocity and associated shear allows the effect of the deformation lift force to be observed when processing blood cells to provide an additional fractionation dimension. In addition, increased velocity enables the integration of upstream Dean focussing of the sample into a confined core stream. This approach collapses inertial transport into a singular horizontal path, eliminating initial positional dissimilarities to increase the efficiency of size-based digital binning. This emphasises the performance gains when moving from sheathless to 2D and then 3D focussing. We further demonstrate that the approach can be extended to spatial binning for resolving a range of biologically relevant sizes and deformation characteristics. Thus we provide experimentally derived quantitative design rules that can be extended to other microparticle and cell sorting scenarios.

## MATERIALS AND METHODS

### Designs

**FIG. 1.**
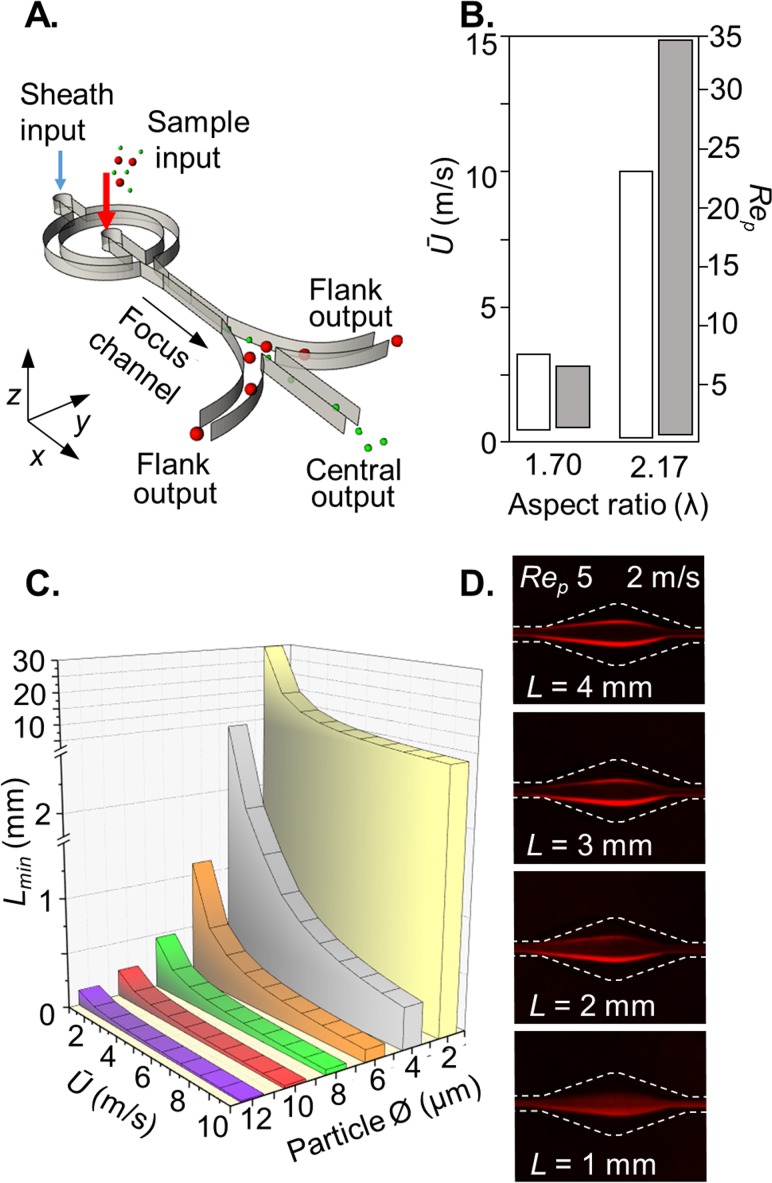
*Dimensional requirements*: (a) inertial microfluidic circuit with hydrodynamic sample focussing into a central column for size-dependent binning. (b) Aspect ratio *λ* comparison; *λ* > 2 greatly extends the velocity (white) and *Re_p_* (grey) dynamic range (see supplementary material, Fig. 2). The *λ* 1.70 experiment involved 50 × 85 *μ*m channel dimensions to focus 15-*μ*m-diameter particles (*a/w* 0.30) and the *λ* 2.17 experiment involved 30 × 65 *μ*m channel dimensions to focus 10-*μ*m-diameter particles (*a/w* 0.33). (c) Theoretical particle size and mean flow velocity scaling effects on channel length requirements.^12^ (d) In practice, a channel focussing length of 2 mm (supplementary material, CAD, design 2) is effective for focussing 10-*μ*m-diameter cell-sized particles.

Inertial focussing devices consisted of a straight, high aspect ratio focussing channel (*w = *30 *μ*m, *h = *65 *μ*m), ending with a gradually expanding region (increasing to 250 *μ*m over a length of 1 mm) that leads to branched outlets. Fluidic resistors at the outlets ensured equal distribution of flow between the outlets. An upstream hydrodynamic lateral sheath flow (*Q_sheath_:Q_sample_*; 2:1) was used to introduce the sample as a central column, with only those particles and cells experiencing sufficient inertial focussing being diverted to the flanking outlets [see Fig. 1(a)]. The optimal focussing channel length was determined using a channel with periodic expansions every 0.5 mm to a width of 150 *μ*m (supplementary material, computer-aided design (CAD), design 1), sufficient to visualise focussing. Subsequent experiments used a focus channel length of 2 mm (supplementary material, CAD, design 2). The 3D Dean focussing element used a 90° curved input channel with a 240 *μ*m average radius and 80 *μ*m width (supplementary material, CAD, design 3). A 7:1 sheath flow for Dean focussing was used in advance of a 1:1 sheath flow for lateral, hydrodynamic focussing. A 7-outlet circuit with a Dean focussing element integrated upstream of the 2 mm inertial focussing channel was used to evaluate the feasibility of multiscale sorting (supplementary material, CAD, design 4).

### Fabrication, assembly, and operation

The inertial focussing device masters were fabricated by standard SU-8 lithography and replicated in poly(dimethyl)siloxane (PDMS, Sylgard 184, Dow Corning). Inlet and outlet ports were prepared using 1 mm Miltex biopsy punches (Williams Medical Supplies Ltd., Gwent, UK) and devices were bonded to glass microscope slides using a 30 s oxygen plasma treatment followed by a 24 h post bake at 60 °C. Inlets were interfaced using Tygon™ tubing (1.09 mm outer diameter, 0.38 mm inner diameter) to Chemyx laboratory syringe pumps and outlets to collection tubes. The focussing conditions are described in terms of the mean fluid velocity, *Ū*.

### Cell isolation

For isolation of red blood cells (RBCs), white blood cells (WBCs), and platelets, human blood was collected by venipuncture from healthy consenting volunteers into Plastic Citrate Vacutainer™ tubes (BD medical) and subsequently separated into RBC, WBC, and platelet fractions: tubes were centrifuged at 1000 rpm for 10 min at room temperature. The buffy coat interface between RBC and plasma layers containing the total WBC population was washed with ice cold water to lyse contaminating RBCs before restoring to an isotonic state using 10× phosphate buffered saline (PBS) followed by centrifugation at 1000 rpm for 10 min. WBCs were re-suspended in 1× PBS at 1 × 10^6^ cell/ml. The RBC layer was collected and diluted in 1× PBS to the original volume before washing via centrifugation then diluted 100-fold (∼5 × 10^7^/ml) for inertial focussing experiments. The platelet rich plasma layer was collected and diluted to 4 × 10^6^/ml. To evaluate focussing of a lymphocyte WBC sub-population, human CD3 T cells (8.6 ± 1.1 *μ*m) were isolated from blood cones using the EasySep™ Human T Cell Isolation Kit (STEMCELL Technologies) following the manufacturer's instructions and used at 1 × 10^6^ cell/ml.

### Imaging

High-speed microscopy was used to record particle and cell distributions. Mirrored positions are combined and presented as normalised half channel width positions ranging from the channel centre line (0.0) to the channel wall (0.5). To quantify particle and cell positions, a high-speed camera (Phantom Miro eX2) was used to capture 4000 fps with a 10 *μ*s exposure, sufficient to determine positions in the 0.1–6.0 m/s mean flow velocity range. Positional data from 100 particles or cells were plotted either as violin plots or distribution curves. Particle distribution was visualised in an Epanechnikov kernel density plot calculated using an R-based script with a smoothing bandwidth of 5. The trajectory of different sized fluorescent polystyrene particles (*ρ* 1.05; Sigma-Aldrich, Kisker Biotech and Thermo Fisher) were visualised using long-exposure (3 s) fluorescent micrographs at the expansion region, using an Olympus CKX41 inverted microscope coupled to a QI Click camera controlled by the open source Micro-Manager software (NIH). Line plot profiles were obtained using Image J and plotted as normalised full channel width (+0.5 to −0.5).

### Focussing efficiency

Particle binning performance was quantified using an Accuri C6 Flow Cytometer (BD Biosciences). Particles were identified by gating on forward and side scatter properties, while cellular fractions were stained with either CellTracker Green CMFDA (Thermo Fisher Scientific) or CellTracker Red CMTPX (Thermo Fisher Scientific) at 1 *μ*M for 30 min prior to inertial flow experiments for subsequent cytometry measurements using fluorescent and scatter characteristics. Cytometry count data was expressed in terms of focussing efficiency, *FE* (%) *= (output flanks/output total) ×* 100.

### Confocal flow imaging

To visualise 2D hydrodynamic and 3D Dean focussing, sample streams were doped with 10 *μ*M fluorescein sodium salt in 1 × PBS. To locate the input flow relative to the channel walls, the device was stained with 100 *μ*g/ml of the grafted copolymer of poly-*L*-lysine and poly(ethylene glycol) labelled with tetramethylrhodamine (PLL-*g*-PEG TRITC) for 30 min immediately following plasma bonding. Confocal Z stack images were obtained using a Leica SP8 confocal microscope with 20× objective, pinhole set to 1 airy unit, and a 0.5 *μ*m step. The Huygens Essential software with a theoretical point spread function based on known parameters was used for image deconvolution.

## RESULTS AND DISCUSSION

### Dimensions of investigation

Predicting inertial transport effects is computationally intensive. Nevertheless, early endeavours^1^ have led to the extrapolation of simple analytical equations^2,5^ that can be used to inform entry into inertial microfluidics. With linear focussing channels applied to cellular dimensions, these design rules indicate effective focussing occurs when *a/D_h_* is >0.07, with a channel aspect ratio (*λ = h/w*) of ≥2,^1,9^ with a particle size-dependent focussing channel length description and within a finite *Re_p_* range of 1–5.^2,5^ Using the *a/D_h_* > 0.07 assumption and the use of human blood as a model system to evaluate sorting, we fabricated 30-*μ*m-wide inertial focussing channels with *λ* ≈ 2.

### 2D hydrodynamic focussing

With purity being a critical performance metric, we have implemented the inertial fractionation circuit shown in Fig. 1(a) with upstream hydrodynamic focussing to confine the sample stream to a central column with subsequent inertial focussing translating particles into outer sheath streams and towards the flanking outlets. Without hydrodynamic focussing, a large proportion of the particles are introduced within streamlines at or near to separation paths irrespective of size (supplementary material, Fig. 1). Consequently, without upstream hydrodynamic focussing, fractionation purity is confounded by the emergence of small, poorly focussed particles from the flank outlets.

### Channel aspect ratio impacts the dynamic range

In conventional planar microfluidic systems, a channel aspect (*λ = h/w*) >1 is required to reduce focussing to the vertical 3^rd^ and 4^th^ equilibria positions that decrease sorting efficiency. We investigated sensitivity to the aspect ratio using 10-*μ*m-diameter particles to identify mean flow velocities where two lateral inertial focussing positions emerge and become stable before the emergence of the two vertical wall positions. This indicates the available *Ū* and associated *Re_p_* dynamic range for processing blood cells. In a simple experiment with analysis by fluorescent imaging, we compared similar *λ* < 2 (1.70) with *λ* > 2 (2.17): As documented in Fig. 1(b) and supplemented with imaging data (supplementary material, Fig. 2), *λ*1.70 provides a dynamic range of *Re_p_* 1.0–4.8 (*Ū* 0.6–3.0 m/s; *Re* 38–188), whereas with *λ*2.17, this was broadened 10-fold to *Re_p_* 0.5–24 (*Ū* 0.2–10 m/s; *Re* 8–409). Thus, the channel aspect ratio is a critical parameter,^10^ confirming the need for *λ* > 2 to attain higher throughput^11^ and the ability to operate at widely ranging velocities.^9^

**FIG. 2.**
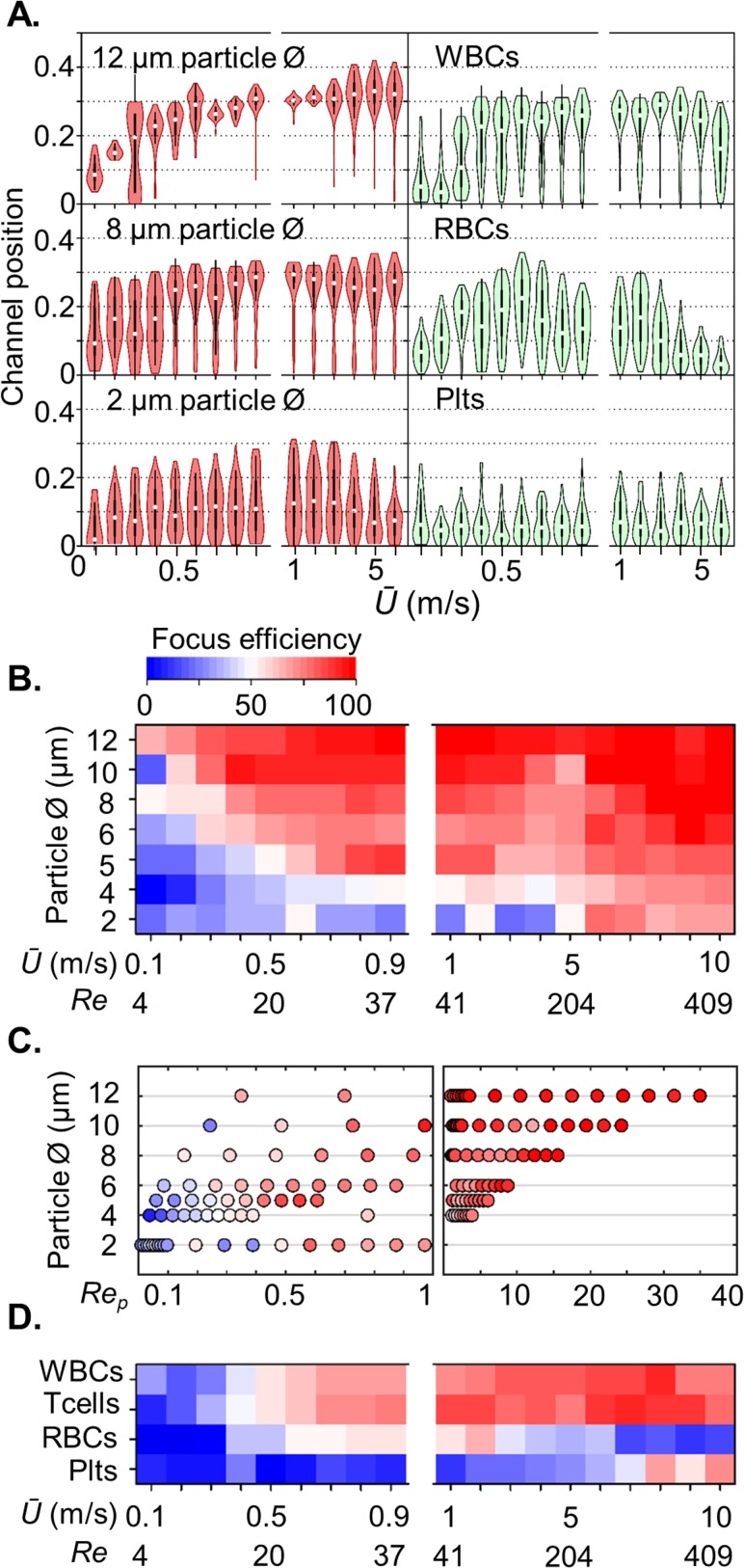
*Inertial focussing of particles and blood cells:* mirrored equilibrium position as a function of mean flow velocity for 12, 8, and 2-*μ*m-diameter particles (red) and blood components (green); WBCs, RBCs, and platelets (a). Heat maps showing the size-dependent velocity (b) and *Re_p_* (c) regimes for efficient particle binning (focussing to flanking outlets, or exiting the central outlet without focussing), coupled with the velocity regime for blood cells (d). Efficient focussing occurs with ≥5-*μ*m-diameter particles. T cells and other white blood cells are effectively separated at *Ū* ≥ 1 m/s, whereas red blood cells and platelets do not migrate to the flanking outlets. The impact of a deformation lift force directing cells towards the channel centre is evident at *Ū* 6 m/s with WBCs and at *Ū* > 2 m/s with RBCs.

### Focal length description

Subsequently, we investigated the size-dependent focal length^2,12^ described by

$$L_{min}=\frac{3\pi \mu }{2\rho \bar{Ū}}\;{\left(\frac{Lc}{a}\right)}^{3},$$

where *L_min_* is the minimum focal length (m), *μ* is the dynamic viscosity (N s/m^2^), *L_C_* is the characteristic channel length, the smallest channel dimension, *ρ* the fluid density (kg/m^3^), *Ū* is the average fluid velocity (m/s), and *a* is the particle diameter. The focussing channel length scales with *1*/*a^3^*, predicting sub-millimetre lengths at 2 m/s for ≥6-*μ*m-diameter particles [Fig. 1(c)]. We tested this using 10-*μ*m-diameter particles in a channel with periodic tapered expansions (supplementary material, CAD, design 1) to provide imaging windows without introducing Dean vortices. In practice, a 2 mm length is required [Fig. 1(d)] and was therefore chosen to evaluate the fractionation cut-off for cell-sized objects, such as the isolation of white blood cells (WBCs) from red blood cells (RBCs) and platelets. This length is 10-fold greater than the predicted length minima for 10-*μ*m-diameter particles. This highlights the varied transport paths produced for particles introduced throughout the channel height and the substantial increases in channel length required to effectively focus the majority of the particle population. Nevertheless, the 2 mm length does not suffer the high pressure drop penalty of lengthy channels (e.g., 40 mm) needed to attain steady-state positions.

### Size- and velocity-dependent inertial focussing

A scaling experiment involving particle diameters ranging from 2 to 12 *μ*m and *Ū* ranging from 0.1 to 10 m/s was used to determine size-dependent focal positions and subsequently the digital binning size threshold (centre or flanking outlets). Larger particles at high, ≥1 m/s, velocities focus to inertial equilibrium positions closer to the channel wall than smaller particles [Fig. 2(a)]. At still higher velocities, suitable for high throughput processing (>10 kHz), the equilibrium position is unperturbed. Despite evident size and velocity scaling, digital binning produced a substantially graded fractionation efficiency, without a distinct size-dependent cut-off [Fig. 2(b)]. Nevertheless, this data set serves to support the general requirement for *a/Dh *≥* *0.07 for appreciable focussing at moderate velocities, channel lengths, and associated practical pressure drops. However, at high *Ū* (≥5 m/s), we have demonstrated that focussing can be achieved at *a/D_h_* values less than 0.07 (e.g., 0.048 for 2-*μ*m-diameter particles). Similarly, longer channels (e.g., >40 mm) can be used to ensure focussing positions reflect the steady state where the *a/D_h_* ratio can also be extended to ∼0.06, with *Re_p_* < 0.1 for small particles.^12^ Our data also shows that for particles with diameters ≥5 *μ*m, the *Re_p_* range for focussing is substantially expanded beyond the generally considered *Re_p_* 1–5 range for inertial focussing [Fig. 2(c)]. In the context of digital binning, this limits size discrimination at high throughput flow rates.

### Blood cell focussing and deformation

Extending the experiment to blood cell preparations, the same size and velocity dependent graded fractionation pattern was observed, with reasonable discrimination of white blood cells (WBCs) from smaller red blood cells (RBCs) and platelets (Plts) at 1 m/s mean flow velocities [Figs. 2(a) and 2(d)]. This demonstrates that polystyrene particles are appropriate models for understanding the inertial transport of spherical cells. However, using the particles as size calibrants, the apparent diameter of RBCs is 4 *μ*m, between the 8-*μ*m-diameter and the 2.5-*μ*m-thickness dimensions. This is not a result of dimension averaging by rotation as the cell tumbles through the vertical axis. Instead, the high shear conditions cause RBCs to align such that the discoid axis is parallel to the channel wall.^11,13^ This predicts an effective diameter of 8 *μ*m, but instead a deformation induced lift force^13–16^ directs the RBCs towards the channel centre, thereby further reducing the distribution of RBCs to the flank outlets. At higher *Ū* (e.g., 6 m/s) enabled using *λ* > 2, the RBCs almost exclusively exit the central outlet [Fig. 2(d)], revealing the highly deformable character^17,18^ of RBCs and confirms previous observations of deformation during inertial transport in linear channels.^11^ These velocities and associated shear conditions exceed those previously investigated and without nanosecond exposure imaging it cannot be excluded that other shape and deformation effects exist. Like RBCs, platelets are typically discoid-shaped with a diameter of 2.0 *μ*m, too small (*a/w* 0.07) for effective focussing in the current system. However, at extreme *Ū* (≥7 m/s), modest focussing occurs [Fig. 2(d)], indicating the rigid nature of platelets, a possible consequence of abundant alpha granules and dense bodies.

White blood cells are less deformable, yet still experience deformation induced lift at the higher mean flow velocities [6 m/s, Fig. 2(a)]. By extending the velocity range without introducing vertical equilibria positions, our system can deliver shear rates (*γ*) and stresses (*σ*) (*Ū* 1–10 m/s; 10^5^–10^6^ s^−1^, and 10^2^–10^3^ Pa) up to 7-fold larger than those reported by Hur *et al.* using a similar linear arrangement.^15^ Together, these results demonstrate the feasibility to measure cell deformation and use this as a sorting index for discriminating similar-sized cells with differing deformation characteristics. Beyond basic research, this is applicable to the diagnosis of cancer,^15,19–21^ inflammation,^22^ blood diseases, and infections, and also for the enrichment of human embryonic stem cells.^22^ For application in regenerative medicine, it is important to note that the inertial transport conditions do not impact cell viability.^13^ We have corroborated this in preliminary experiments involving the sorting and harvesting of cell lines and primary cells.

To investigate the origins of the graded fractionation efficiency, the particle size standards were imaged, revealing broader than anticipated size distributions (coefficient of variation (CV) 11.7%–22.8%), larger than the different blood cell fractions (CV 5.9%–17.7%; supplementary material, Fig. 3). An additional and key factor we reasoned was the varied entry position of particles and cells. Even with hydrodynamic focussing into a vertical sample column, multiple and complex inertial transport paths exist, each with a distinct force-position diagram resulting in dissimilar transport conditions and the resultant graded fractionation efficiency. The simplest solution entails introducing the sample as a tightly confined core stream that is positioned at the channel mid-height and mid-width.

**FIG. 3.**
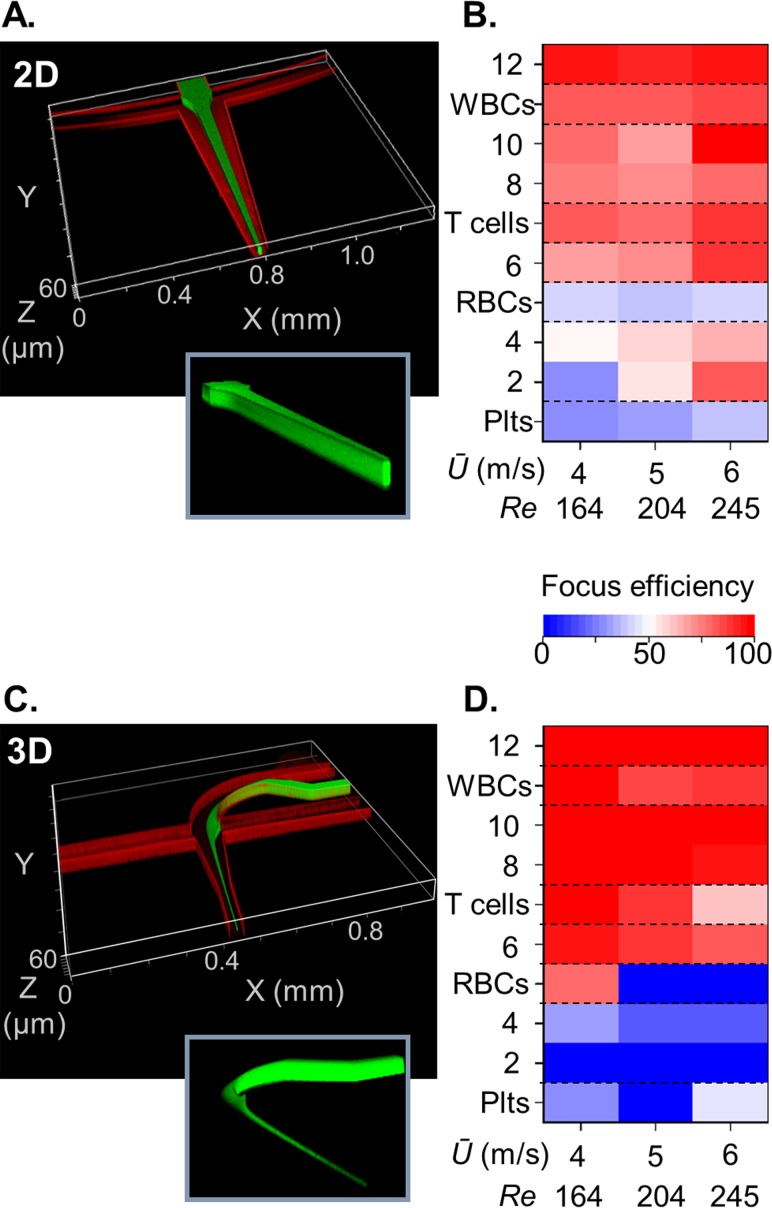
*Upstream 3D sample focussing produces a distinct fractionation cut-off.* (a) Hydrodynamic focussing of the sample into a central column prior to inertial focussing and (b) produces graded size-dependent particle and cell sorting efficiencies with *Ū* 4–6 m/s. (c) Upstream Dean and hydrodynamic sample focussing into a tight core stream (d) produces a 4-*μ*m-diameter sorting cut-off with *Ū* 4–6 m/s. Insets, structured sample streams without microchannel walls.

### Dean focussing sample streams

Core sample flows can be produced using multi-layered channel arrangements.^23–25^ Instead, we chose to avoid complex fabrication and assembly processes and exploit the extended *Re_p_* to access upstream velocity regimes required to introduce counter-rotating Dean flows to vertically confine the sample flow in advance of lateral confinement by hydrodynamic focussing. This 3D hydrodynamic focussing technique was demonstrated by Huang *et al.* using a 90° curved channel, with a sheath-sample flow ratio of ∼7 and with a Reynolds number of 74 to produce the necessary Dean number *κ* of 43, as defined by *κ = Re·(Dh/R)^1/^*^2^, where *R* is the channel radius of curvature.^26–28^ Using similar dimensions, we interfaced this Dean focussing geometry upstream of the inertial focussing element and investigated the velocity effects (supplementary material, CAD, design 3). The sample stream enters the curved channel, with *R *=* *240 *μ*m, and combines with the sheath flow producing co-parallel flows. At appreciable velocities, the sample stream is stretched by the secondary flow, across the 80 *μ*m channel width while being compressed into a thin sheet at the channel mid-height (see the supplementary material, Fig. 4). Near full channel width sample extension was achieved at *κ* > 30 and is required to centre the sample stream and thus avoid bias to the outlets. Subsequent combination with flanking sheath flows produced an elliptical sample core, 7.5 *μ*m wide and 12.0 *μ*m high (Fig. 3). While continuously curved spiral channels can be used to fractionate particles^29^ and cells,^30^ short length (<400 *μ*m) Dean focussing is insadequate for size-based sorting. Instead, the sample stream containing particles and cells is focussed to a common position for unbiased linear inertial fractionation. Within the narrow, 30-*μ*m-wide, inertial focussing channel, this produces a ≥4 m/s mean flow velocity. A >2 channel aspect ratio is therefore required to prevent the development of the second, centre top and bottom, equilibria pair, and associated losses in fractionation efficiency.

**FIG. 4.**
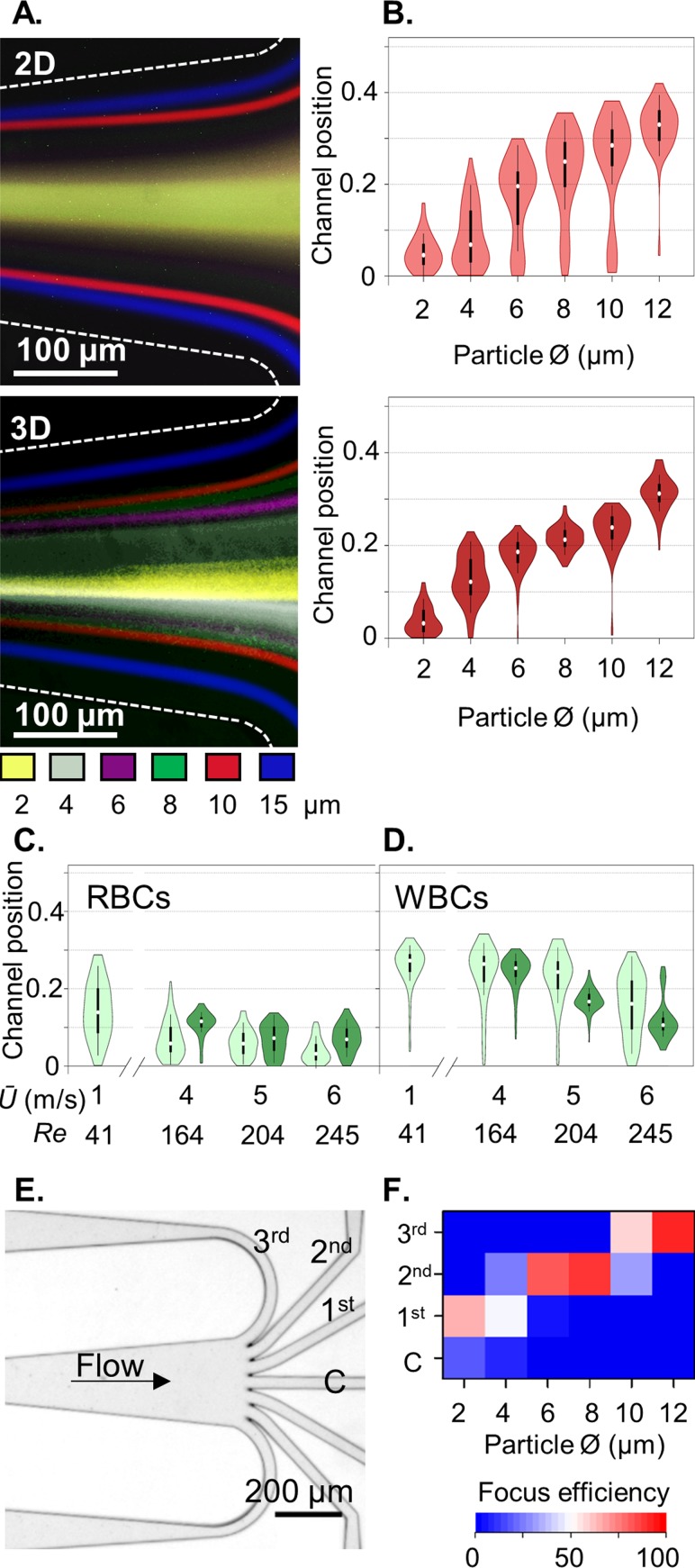
*Upstream 3D focussing improves the spatial resolution of inertial focussing*: (a) long-exposure fluorescent image overlays of 2–12 *μ*m fluorescent particles at the outlets of 2D and 3D inertial focussing devices and (b) corresponding outlet channel positions (*n *=* *100, 2D light red, 3D dark red). (c) Deformation effects on RBCs and WBCs at high *Ū* in 2D (light green) and 3D (dark green) inertial focussing. (e) A 7-outlet system and (f) resulting fractionation efficiency using 3D focussing at *Ū* 5 m/s.

### A single inertial focussing path improves the fractionation efficiency

Unlike lengthy spiral systems for differential particle focussing at low Dean numbers, Dean focussing over short transport lengths with high curvature and associated Dean numbers acts to deliver all particles, irrespective of size, to a single fluid core within the channel cross section. This creates a common origin in advance of linear inertial focussing. To evaluate the benefits of upstream Dean-driven 3D sample focussing, fractionation efficiencies of the particle standards and blood cells were compared with those from the 2D sample focussing technique (Fig. 3). 3D focussing produces a distinct size-based cut-off between 6.27 *μ*m (95%) and 4.10 *μ*m (32%) particles at 4 m/s, whereas with 2D focussing, the efficiency was only 70% and 53%, respectively. Extending to blood cell fractionation, 3D focussing increased T cell focussing (99%) as compared to 2D focussing (82%) and limited RBC focussing from 42% to 0.3% at *Ū* 6 m/s.

Improved purity is a consequence of restricting the input position to the channel mid-height and mid-width, thus collapsing the transport path to a single force diagram. With 3D focussing and a 2 mm separation length, particles with diameters ≥0.15*w* attain the steady state focal position. In comparison, 2D focussing involves numerous transport paths, with many of these requiring longer inertial focussing channels. As a consequence, not all particles attain long wall equilibrium positions, whilst at high *Ū* (>4 m/s), particles proximal to the short wall equilibrium positions can attain 3^rd^ and 4^th^ equilibrium positions. Overall, these features create a kinematic fractionation system with graded performance. Thus the efficiency gains and enhanced purity confirm the merits of integrating a 3D focussing element within the microfluidic circuit.

### A single inertial focussing path increases the spatial resolution

The complex force variations involved in inertial focussing prevent the numerical prediction of the equilibrium positions.^6^ To address this knowledge gap, we next investigated the effects of 3D focussing on the downstream inertial equilibrium positions. Long-exposure fluorescent imaging and high speed imaging data documented in Figs. 4(a) and 4(b) and supplementary material, Fig. 5 demonstrate that upstream 3D focussing improves particle focussing, whereas 2D focussing typically produces a trimodal particle distribution, with mirrored lateral focussing positions. Furthermore, with 3D focussing, the channel position distributions now closely reflect the particle size distributions (see supplementary material, Figs. 3 and 5), demonstrating an exclusive size-dependent separation mode with rigid particles. By providing a 3D focussed sample input, the central positions are removed and 4-*μ*m-diameter particle focussing can now be observed. The same improved focussing is evident with blood cells [Figs. 4(c) and 4(d)]. Using full width half maxima (FWHM) analysis, the inertial focussing improvement with upstream 3D Dean focussing is modest (supplementary material, Fig. 5). Importantly, 3D focussing prevents proximity to 3^rd^ and 4^th^ equilibria during entry to the inertial focussing channel to ensure only lateral focussing to the 1^st^ and 2^nd^ equilibria positions, resulting in large gains in focussing efficiency. Thus by constraining the sample input as a core, particle and cellular separations are enhanced. Shown in Fig. 4(d), WBC deformation responses are more focussed when introduced as a sample core. For optimal multiscale sorting, further efforts are required to increase peak-to-peak separation. The improvements in spatial resolution with upstream 3D focussing open the possibility for multimodal inertial fractionation. As a first demonstration of spatial binning for multiplexed fractionation, a symmetric radial array with seven equal fluidic resistance exit paths was interfaced to the end of the inertial focussing channel [Fig. 4(e), supplementary material, CAD, design 4]. In this example, 12-*μ*m-diameter particles are efficiently (93%) focussed to the outer channel pair (3^rd^ exits), 8.1 and 6.0-*μ*m-diameter particles to the neighbouring channels (2^nd^ exits, 88% and 82% efficiency, respectively), and 2.2-*μ*m-diameter particles moderately (67%) to the inner channel pair [1^st^ exits; Fig. 4(f)]. Future circuits can be designed with the known particle and cell focussing positions in mind and with increased channel expansion to amplify the separation distance between the different particle streams.^11,31^ Beyond this, the approach can be hyphenated with sequential inertial focussing elements^32,33^ to increase the fractionation efficiency.

## CONCLUSIONS

In summary, we have used an experimental approach to systematically explore the inertial focussing conditions appropriate for the resolution of whole blood cell fractions. In this manner, we identified sensitivity to the channel aspect ratio and used this to extend the effective velocity range and enable the integration of an upstream Dean focussing element to produce a confined sample core stream. This produces a single, mirrored, inertial transport path, thereby reducing the varied and complex inertial transport phenomena to a function of size and deformation, resulting in precision manipulations and gains in fractionation efficiency. The ability to access higher velocity flow regimes while restricting focussing lengths and associated pressure drops to tolerable regimes invites the possibility to scale the microfluidic circuit for the enrichment and discrimination of microparticle and exosomes to access their diagnostic potential. Overall, we provide a generalised framework for the straightforward design of inertial microfluidic fractionation systems for resolving cellular and sub-cellular samples.

## SUPPLEMENTARY MATERIAL

See supplementary material for graphical illustration of the microfluidic concept for resolving blood cell fractions. Inertial focussing without hydrodynamic sheath confinement (Fig. 1). Sensitivity to the channel aspect ratio (Fig. 2). Particle and cell diameter distributions (Fig. 3). Dean sample flow focussing velocity requirements (Fig. 4). Introducing particles and cells as a fluidic core improves the resolving power of inertial fractionation (Fig. 5). Design file for the microfluidic circuits (CAD).
